# Severe hospital-acquired hyponatremia in acutely ill children receiving moderately hypotonic fluids

**DOI:** 10.1007/s00467-021-05227-0

**Published:** 2021-08-16

**Authors:** Saara Lehtiranta, Minna Honkila, Merja Kallio, Kimmo Halt, Niko Paalanne, Tytti Pokka, Terhi Tapiainen

**Affiliations:** 1 Department of Pediatrics and Adolescent Medicine, Oulu University Hospital, PO Box 23, N90029 OYS, Oulu, Finland; 2 PEDEGO (Pediatrics, Dermatology, Gynecology and Obstetrics) Research Unit and Medical Research Centre Oulu, University of Oulu, Oulu, Finland; 3 Department of Pediatric Cardiology, New Children’s Hospital, University Hospital of Helsinki, Helsinki, Finland; 4 Biocenter Oulu, University of Oulu, Oulu, Finland

**Keywords:** Hyponatremia, Electrolyte disorder, Maintenance fluid therapy, Isotonic fluid therapy

## Abstract

**Background:**

Hypotonic fluids have been associated with hospital-acquired hyponatremia. The incidence of life-threatening severe hyponatremia associated with hypotonic fluids has not been evaluated.

**Methods:**

This was a population-based cohort study of 46,518 acutely ill children 15 years of age or under who visited the pediatric emergency department (ED) at Oulu University Hospital, Finland, between 2007 and 2017. We retrieved all electrolyte measurements from the comprehensive electronic laboratory system and reviewed medical records for all patients with severe hyponatremia.

**Results:**

The overall occurrence of severe hyponatremia (serum sodium < 125 mmol/L) was found in 27 out of 46,518 acutely ill children (0.06%, 95% confidence interval 0.04–0.08%). After admission, severe hyponatremia developed in seven of 6,984 children receiving moderately hypotonic fluid therapy (0.1%, 95% confidence interval 0.04–0.2%), usually within 8 h of admission. All children who developed severe hyponatremia during hospitalization were severely ill.

**Conclusion:**

In this register-based cohort study of children presenting to the ED, severe hyponatremia developed in one of 998 acutely ill children receiving moderately hypotonic fluid therapy.

**Graphical abstract:**

A higher resolution version of the Graphical abstract is available as Supplementary information 
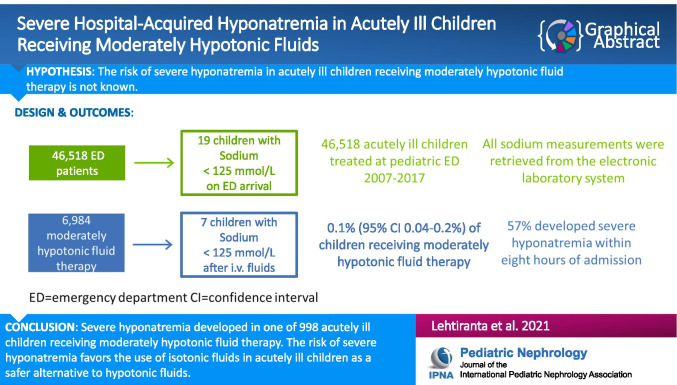

**Supplementary Information:**

The online version contains supplementary material available at 10.1007/s00467-021-05227-0.

## Introduction

Several well-conducted randomized controlled trials have shown that isotonic fluids are effective in preventing the development of mild or moderate hyponatremia in acutely ill children receiving maintenance fluid therapy [1–4]. Accordingly, the American Academy of Pediatrics (AAP) has recommended the use of isotonic maintenance fluid therapy to prevent hyponatremia in children [1]. The risk of developing severe hyponatremia is not known in children receiving moderately hypotonic fluid therapy. Furthermore, there are limited data on the overall occurrence and outcome of severe hyponatremia in acutely ill children [1].

In the present study, we investigated the occurrence of hospital-acquired severe hyponatremia in children presenting to the pediatric emergency department (ED) and receiving moderately hypotonic fluid therapy in a population-based cohort of 46,518 children. In addition, we reported the occurrence of sodium abnormalities in acutely ill children.

## Methods

### Study design

This was a register-based, population-based cohort study in acutely ill children presenting to the pediatric ED. The research protocol was approved by the Ethics Committee of Human Sciences at the University of Oulu, Finland. According to Finnish legislation, individual informed consent is not required for register-based medical research. All data were collected and analyzed according to current data protection requirements and legislation in the European Union.

All acutely ill children aged 15 years and under who were treated at the pediatric emergency department (ED) of Oulu University Hospital, Finland, between 2007 and 2017 were included in the study. Oulu University Hospital is the only hospital with a pediatric ED and with pediatric wards in a catchment area of approximately 92,000 children. No surgical trauma patients were treated at the pediatric ED in our hospital during the study. Patients with ongoing oncological treatment were also excluded.

### Definitions of hyponatremia

The following definitions were used for hyponatremia, according to the lowest serum sodium concentration levels: mild, 130–134 mmol/L; moderate, 125–129 mmol/L; and severe hyponatremia, < 125 mmol/L. We included all patients who presented with hyponatremia at the pediatric ED or who developed hyponatremia within 7 days after admission to the ED, during hospitalization.

### Data collection

All the sodium values that were examined at the pediatric ED or after admission to the wards for general pediatrics, pediatric infectious diseases, pediatric neurology, and pediatric surgery, or the pediatric intensive care unit (PICU) were retrieved and inserted into the study database from the hospital’s comprehensive electronic laboratory system. During the study period, moderately hypotonic maintenance fluid therapy with 60–80 mmol/L of sodium was routinely used. Medical records were fully reviewed in all patients with severe hyponatremia. In addition, we reviewed all medical records, including the use of intravenous fluid therapy, for a random sample of 801 acutely ill children from a population of 11,753 children with electrolyte measurements. In this sample of 801 children, 476 (59%) had received intravenous fluid therapy. From these data, we estimated the proportion of children that had received intravenous fluid therapy during hospitalization and used it as an additional denominator in the analyses.

### Statistical analysis

We calculated the proportions of children with mild, moderate, and severe hyponatremia in all children visiting the ED, in children with electrolyte measurements at the ED, and in children who had received intravenous fluid therapy. We calculated the 95% confidence intervals (CI) of the proportions. For population-based incidence calculations, the annual number of children and adolescents 15 years old or younger living in the catchment area was retrieved from the national Statistics Finland register (www.stat.fi).

## Results

**Fig. 1:**
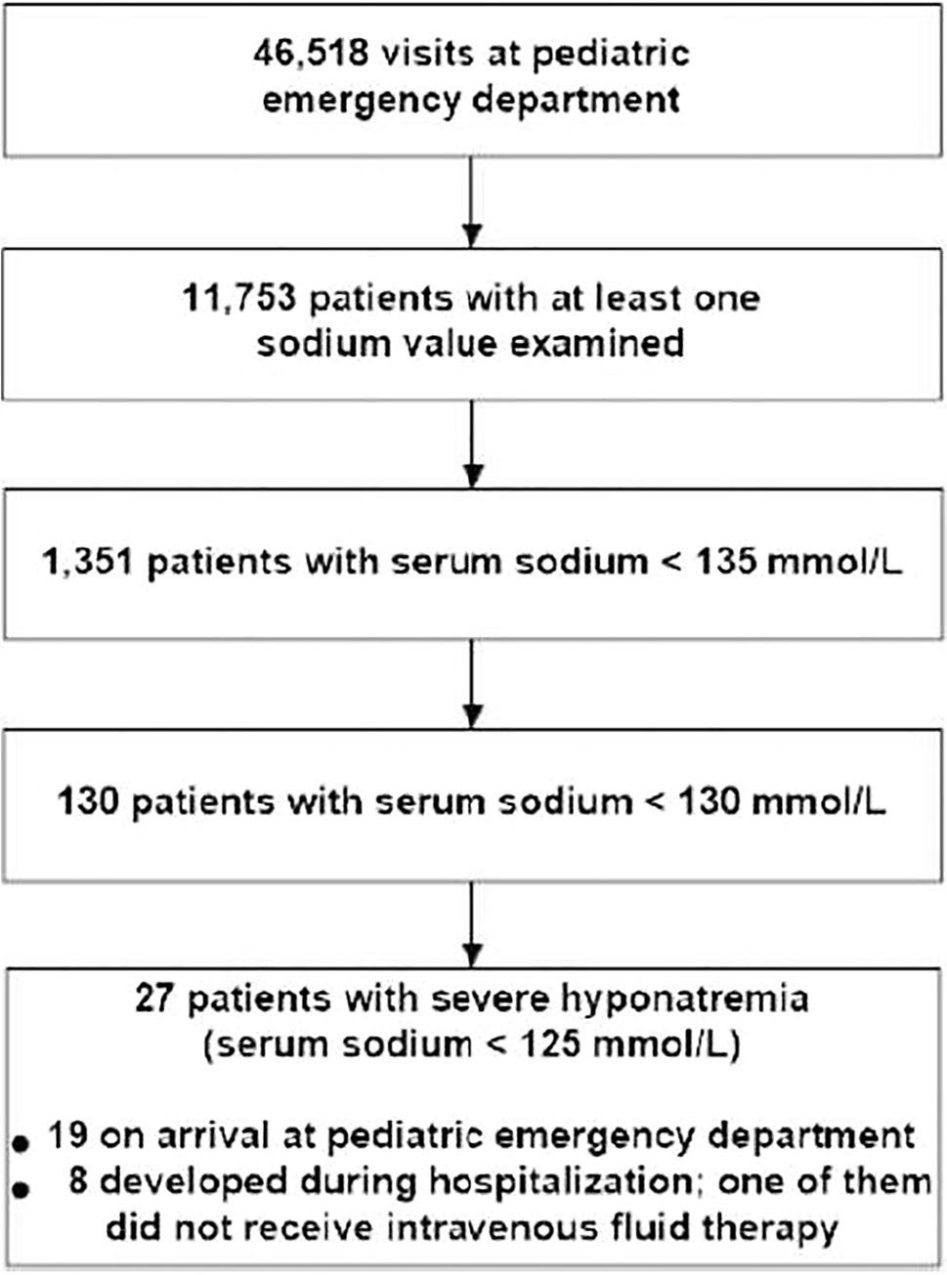
Study profile

There were 46,518 visits at the pediatric ED during the 10-year study period from 2007 to 2017 (Fig. 1). At least one sodium measurement was performed in 11,753 (25%) acutely ill children. By using the random sample of 801 patients, we estimated that 6,984 children (15%) had received intravenous fluid therapy during hospitalization. All patients treated with intravenous maintenance fluid therapy after admission received moderately hypotonic fluids.

A total of 1,351 (2.9%) patients had hyponatremia (serum sodium < 135 mmol/L) (Table 1). Mild hyponatremia (serum sodium 130–134 mmol/L) occurred in 2.6%, moderate hyponatremia (serum sodium 125–129 mmol/L) in 0.2%, and severe hyponatremia (serum sodium < 125 mmol/L) in 0.06% of acutely ill children. Among the 27 acutely ill children with severe hyponatremia, most (19/27, 70%) already had severe hyponatremia on arrival at the ED before receiving intravenous fluid therapy. The annual population-based incidence of severe hyponatremia was 2.9 (95% CI 2.0–4.3) per 100,000.

**Table 1:** Hyponatremia in acutely ill children

Hyponatremia	N	Annual population-based incidence per 100,000 (95% CI)	Hyponatremia in acutely ill children (N = 46,518)		Hyponatremia in children with at least one sodium measurement (N = 11,753)	
			Occurrence per 100,000 (95% CI)	Ratio	Occurrence per 100,000 (95% CI)	Ratio
*All cases*						
< 120 mmol/L	7	0.8 (0.3–1.6)	15 (6–31)	1:6,646	60 (24–123)	1:1,679
120–124 mmol/L	20	2.2 (1.3–3.4)	43 (26–66)	1:2,308	170 (104–263)	1:588
< 125 mmol/L	27	2.9 (2.0–4.3)	58 (38–84)	1:1723	230 (151–334)	1:435
125–129 mmol/L	103	11 (9.1–14)	221 (181–268)	1:452	876 (716–1,062)	1:114
130–134 mmol/L	1,221	133 (125–140)	2,625 (2,481–2,774)	1:38	10,389 (9,842–10,954)	1:10
Hospital-acquired*						
< 125 mmol/L	7	0.8 (0.3–1.6)	15 (6–34)	1:6,646	60 (24–123)	1:1,679

CI, confidence interval; ED, emergency department

^*^During moderately hypotonic (containing 60–80 mmol/L of sodium) intravenous fluid therapy

Severe hyponatremia developed during hospitalization in seven of 6,984 patients (0.1%, 95% CI 0.04–0.2%) who received moderately hypotonic fluids (ratio 1:998) (Table 1, Table 2). In most of these children (4/7, 57%), the condition developed rapidly, within 8 h of admission (Table 2). Most patients with severe hospital-acquired hyponatremia were euvolemic at the ED but had reduced enteral intake of fluids. The suspected mechanism of hospital-acquired hyponatremia was related to inappropriate antidiuretic hormone (ADH) secretion in most patients.

**Table 2:** Patients with severe hospital-acquired hyponatremia (serum sodium < 125 mmol/L)

Case	Sodium at ED (mmol/L)	Time to severe hyponatremia (h)	Lowest sodium (mmol/L)	Intravenous fluid therapy before severe hyponatremia	Management of severe hyponatremia	Outcome
1. 1-year-old female subdural hematoma, first misdiagnosed as gastroenteritis	139	8	122	20 mL/kg ringersteril twice Moderately hypotonic maintenance fluid^*^	Transfer to PICU 3% saline infusion Computed tomography examination of the head	Recovered Normal magnetic resonance imaging of the brain 3 months after surgery Follow-up by pediatric neurologist
2. 7-month-old male acute leukemia, critically ill	137	4	123	Double amount of maintenance fluid by leukemia protocol	Transfer to PICU Modification of iv fluid therapy	Recovered
3. 6-year-old female severe Kawasaki disease	131	8	123	10 mL/kg ringersteril twice Moderately hypotonic maintenance fluid^*^	Transfer to PICU Modification of iv fluid therapy	Recovered
4. 11-month-old male acute tubular necrosis, developed anuria	131	23	123	10 mL/kg ringersteril four times Moderately hypotonic maintenance fluid^*^	Transfer to PICU Restriction of iv fluid therapy Hemodialysis	Recovered Kidney transplantation at the age of 6 years
5. 6-year-old male pleural empyema, immunodeficiency	130	60	123	Moderately hypotonic maintenance fluid^*^	Restriction and modification of iv fluid therapy	Recovered
6. 1-month-old male respiratory syncytial virus bronchiolitis	126	5	124	Moderately hypotonic maintenance fluid^*^	Transfer to PICU Modification of iv fluid therapy Need for invasive ventilation	Recovered Follow-up by pediatric neurologist due to developmental delay
7. 15-year-old female allogeneic HSCT, cystitis due to polyoma virus	Not measured	17	124	Moderately hypotonic maintenance fluid^*^	Modification of iv fluid therapy and 0.9% saline bolus	Recovered

^*^Maintenance fluid containing 60–80 mmol/L of sodium, amount of fluid counted by the Holliday-Segar method HSCT hematopoietic stem cell transplantation

All children who developed severe hospital-acquired hyponatremia while receiving moderately hypotonic fluids were severely ill, and two out of seven children (29%) had neurological symptoms (Table 2). One child with subdural hematoma and severe hyponatremia required neurological follow-up after discharge, and one child with respiratory syncytial virus bronchiolitis and severe hyponatremia was later followed up by a pediatric neurologist due to developmental delay (Table 2).

## Discussion

In this register-based cohort study of acutely ill children presenting to the pediatric ED, the risk of severe hospital-acquired hyponatremia was 0.1% (1:998) in acutely ill children receiving moderately hypotonic fluid therapy. The risk of severe hyponatremia in the present study favors the use of isotonic fluids as a safer alternative to hypotonic fluids for the initiation of intravenous fluid therapy in acutely ill children as recommended by the current AAP clinical practice guideline [1].

We found that severe hyponatremia developed rapidly during hospitalization, within the first 8 h of the fluid therapy in most children. Notably, one critically ill child with acute leukemia, who had normal serum sodium at presentation, developed severe hyponatremia 4 h after receiving moderately hypotonic fluids. This is in accordance with previous case reports of fatal hyponatremia [5–7] and hyponatremic encephalopathy [8] showing that severe hyponatremia commonly occurs during the first 24–48 h of fluid therapy. Our findings support taking active electrolyte measurements to prevent harm in acutely ill children, especially in those who are severely ill, to decrease the risk of iatrogenic severe hyponatremia. The cost–benefit analysis of active electrolyte measurements in all admitted children was not, however, conducted in the present study.

The AAP clinical practice guideline of maintaining intravenous fluids in children excludes patients with neurosurgical disorders, cardiac disease, hepatic disease, cancer, and kidney dysfunction because they are excluded from most randomized trials [1]. The AAP guideline further states that studies in which researchers evaluate optimal fluid management in these groups of patients are necessary. In the present study, among seven children who developed hospital-acquired severe hyponatremia after receiving moderately hypotonic fluids, one child had a subdural hematoma, one had a newly diagnosed cancer, and one had kidney dysfunction. Thus, as the initiation of fluid therapy, moderately hypotonic fluid therapy may not be optimal in these pediatric populations with severe underlying conditions.

In most children in the present study, the development of severe hyponatremia was likely attributable to inappropriate ADH secretion resulting in impaired kidney water excretion. A strong cortisol response, cerebral disease, and pulmonary infection are known risk factors for inappropriate antidiuretic hormone excretion [9, 10]. Other causes of severe hyponatremia observed in our study and in previous studies are water intoxication and kidney failure [11, 12]. Thus, in acutely ill children, understanding the risks of inappropriate ADH secretion and kidney function as well as the risks of excessive free water supply is important in avoiding progressive hyponatremia. In clinical practice, after isotonic correction of volume depletion, it may be reasonable to reduce the total fluid delivery rate rather than to provide high rates of isotonic fluid as maintenance therapy.

Up to 50% of children have been reported to develop neurological symptoms if serum sodium falls below 125 mmol/L [7]. In the present study with a population-based study design, the observed occurrence of neurological symptoms was significantly lower. This may be explained by the selected patient populations in previous studies [13]. Furthermore, the previously reported rate of encephalopathy may have been overestimated as many of the patients with neurological symptoms in previous studies had underlying medical conditions that may partly explain the symptoms.

There are several strengths of this study. This is one of the first large cohort studies investigating the risk of severe hospital-acquired hyponatremia in acutely ill children receiving moderately hypotonic fluid therapy. The comprehensive and fully electronic, web-based laboratory system used in our hospital enabled the high-quality study design. Thus, our study provides a reliable estimate of hyponatremia in a population-based sample of representative acutely ill children. We were able to collect data from original medical records, including details about fluid therapy, in all children with severe hyponatremia. Even though the data regarding the fluid therapy of all 46,000 children were not available electronically, we performed a manual review of full original medical records in a random sample of children to produce a reliable denominator for those receiving fluid therapy.

There are some limitations of this study. This was a single-center study, and the results may not be generalizable to all pediatric populations. Children presenting to the pediatric ED represent only a subset of all pediatric patients. Furthermore, most acutely ill oncological and surgical patients were not included in the study population. As the symptoms of hyponatremic encephalopathy are difficult to recognize [14], this retrospectively analyzed cohort may underestimate the risk of neurological symptoms in children with severe hyponatremia. In addition, surgical trauma patients were not treated at the pediatric ED during the study. Most importantly, the study was an observational cohort study in the era of moderately hypotonic fluid therapy, and we do not know whether isotonic fluid therapy might have been effective in preventing the severe hyponatremia reported in our study. Yet, this is one of the first attempts to produce an estimate for the risk of severe hyponatremia in acutely ill children treated with moderately hypotonic fluid therapy.

In conclusion, in this register-based cohort study of children presenting to a pediatric ED, severe hyponatremia developed in one of 998 acutely ill children receiving moderately hypotonic fluid therapy. In the future, we suggest that the risk of severe hospital-acquired hyponatremia in children receiving isotonic fluid therapy is investigated.

## Supplementary Information

Supplementary file1 (pptx 70.4 KB )

## Data Availability

Data sharing of anonymous data is available from the authors for predefined clinical research purposes.

## References

[1] Feld LG, Neuspiel DR, Foster BA, Leu MG, Garber MD, Austin K, Basu RK, Conway EE, Fehr JJ, Hawkins C, Kaplan RL, Rowe EV, Waseem M, Moritz ML, Subcommittee on fluid and electrolyte therapy,  (2018). Clinical practice guideline: maintenance intravenous fluids in children. Pediatrics.

[2] Torres SF, Iolster T, Schnitzler EJ, Siaba Serrate AJ, Sticco NA, Rocca Rivarola M (2019). Hypotonic and isotonic intravenous maintenance fluids in hospitalised paediatric patients: a randomised controlled trial. BMJ Paediatr Open.

[3] McNab S, Duke T, South M, Babl FE, Lee KJ, Arnup SJ, Young S, Turner H, Davidson A (2015). 140 mmol/L of sodium versus 77 mmol/L of sodium in maintenance intravenous fluid therapy for children in hospital (PIMS): a randomised controlled double-blind trial. Lancet.

[4] Kannan L, Lodha R, Vivekanandhan S, Bagga A, Kabra SK, Kabra M (2010). Intravenous fluid regimen and hyponatraemia among children: a randomized controlled trial. Pediatr Nephrol.

[5] Koczmara C, Hyland S, Greenall J (2009). Hospital-acquired acute hyponatremia and parenteral fluid administration in children. Can J Hosp Pharm.

[6] Grissinger M (2013). Hyponatremia and death in healthy children from plain dextrose and hypotonic saline solutions after surgery. P T.

[7] Moritz ML, Ayus JC (2005). Preventing neurological complications from dysnatremias in children. Pediatr Nephrol.

[8] Arieff AI, Ayus JC, Fraser CL (1992). Hyponatraemia and death or permanent brain damage in healthy children. BMJ.

[9] Anderson RJ, Chung HM, Kluge R, Schrier RW (1985). Hyponatremia: a prospective analysis of its epidemiology and the pathogenetic role of vasopressin. Ann Intern Med.

[10] Jones DP (2018). Syndrome of inappropriate secretion of antidiuretic hormone and hyponatremia. Pediatr Rev.

[11] Levin TL, Abramson SJ, Burbige KA, Connor JP, Ruzal-Shapiro C, Berdon WE (1991). Salt losing nephropathy simulating congenital adrenal hyperplasia in infants with obstructive uropathy and/or vesicoureteral reflux–value of ultrasonography in diagnosis. Pediatr Radiol.

[12] Hauser GJ, Kulick AF (2014) Electrolyte disorders in PICU. In: Wheeler DS, Wong HR, Shanley TP (ed) Pediatric critical care medicine. Vol 3. Gastroenterological, endocrine, renal, hematologic, oncologic and immune systems, 2nd edn. Springer, London, pp 147–171

[13] Halberthal M, Halperin ML, Bohn D (2001). Lesson of the week: acute hyponatraemia in children admitted to hospital: retrospective analysis of factors contributing to its development and resolution. BMJ.

[14] Moritz ML, Ayus JC (2010). New aspects in the pathogenesis, prevention, and treatment of hyponatremic encephalopathy in children. Pediatr Nephrol.

