# Supplementary material for: Severe hospital-acquired hyponatremia in acutely ill children receiving moderately hypotonic fluids
Source: Pediatr Nephrol. 2021 Aug 16;37(2):443–8. doi: 10.1007/s00467-021-05227-0 (PMC8816776; doi:10.1007/s00467-021-05227-0)
Supplement: Supplementary file 1 — Supplementary file1 (pptx 70.4 KB ) [file 467_2021_5227_MOESM1_ESM.pptx]

## Slide 1
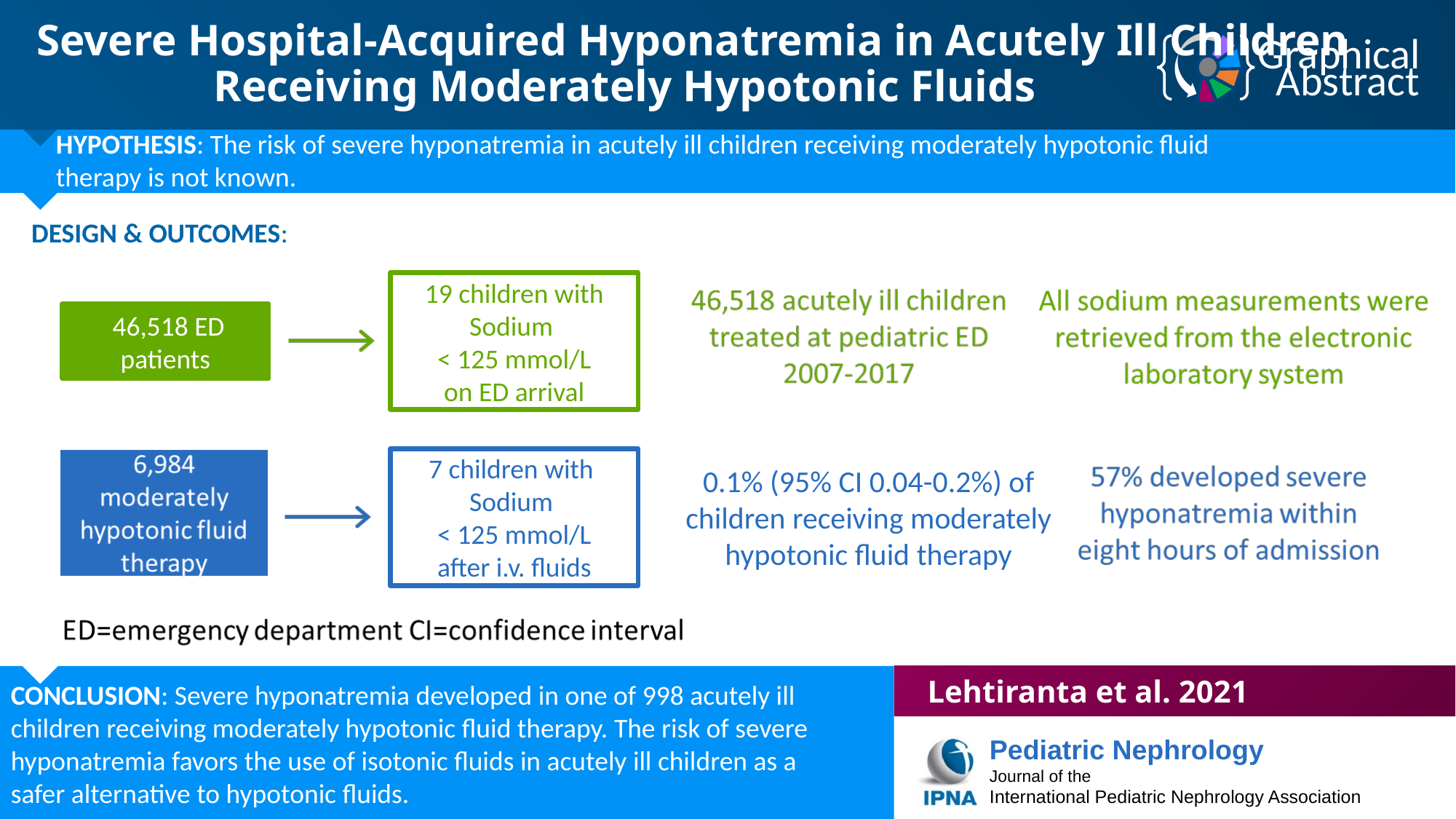

Severe Hospital-Acquired Hyponatremia in Acutely Ill Children Receiving Moderately Hypotonic Fluids
HYPOTHESIS: The risk of severe hyponatremia in acutely ill children receiving moderately hypotonic fluid therapy is not known.
DESIGN & OUTCOMES:
19 children with Sodium
< 125 mmol/L
on ED arrival
 46,518 ED patients
7 children with
Sodium
< 125 mmol/L
after i.v. fluids
0.1% (95% CI 0.04-0.2%) of children receiving moderately hypotonic fluid therapy
Lehtiranta et al. 2021
CONCLUSION: Severe hyponatremia developed in one of 998 acutely ill children receiving moderately hypotonic fluid therapy. The risk of severe hyponatremia favors the use of isotonic fluids in acutely ill children as a safer alternative to hypotonic fluids.
